# Supplementary material for: Chromothripsis during telomere crisis is independent of NHEJ, and consistent with a replicative origin
Source: Genome Res. 2019 May;29(5):737–49. doi: 10.1101/gr.240705.118 (PMC6499312; doi:10.1101/gr.240705.118)
Supplement: Supplemental Material [file supp_gr.240705.118_Supplemental_file_1.zip › contigs/annotated_contigs/DB79/contig.3.DB79_length_409_mean_cov_4.76772616137.docx]

**DB79_length_409_mean_cov_4.76772616137**

```
AGAATCAGTATCACTTGCATTAGAGAAGGGGGAGGGTAGGTGGCCATATGGTGGGAGGGGAT|GCATGTTAATGAAGTAAA|TCCCTCT
 >chr8:68492392-68492454 - E=9e-26 p=3e-02                                       >chr9:
GGGGGTGACTAGAACCGCTAGGCAACAGGGTACACAGCAGGGCAGAGTGCTGCATGAGCAGCCAGGAGTGGGCCTGCAGCCAGCCCAGG
131855664-131855870 - E=2e-109
CAGGACAGGGAGGGGAGAGCGAACAGTCAAGAGGCTGCCAGACAGACACCCTGGATTCCCAAAACTCGCCTAAGAACCCTACAGGTCTG

AATATAAGGTTGGATTTTTTT|CTGTATTTTGTAATTCCCTCAATGAATTTTTCACTTGCAGAACTTCTATTTTATTATTTAAAATAAT
                       >chr2:52805961-52806084 + E=2e-62
AACTGTCTCTTCTTTTATATCCCAAATTGTGTTTTTTTTTCTAATTTATATGTGTT
```
